# Presurgical Evaluation of Epilepsy Using Resting-State MEG Functional Connectivity

**DOI:** 10.3389/fnhum.2021.649074

**Published:** 2021-07-02

**Authors:** Na Xu, Wei Shan, Jing Qi, Jianping Wu, Qun Wang

**Affiliations:** ^1^Department of Neurology, Beijing Tiantan Hospital, Capital Medical University, Beijing, China; ^2^National Clinical Research Center for Neurological Diseases, Beijing, China; ^3^Advanced Innovation Center for Human Brain Protection, Capital Medical University, Beijing, China; ^4^Beijing Institute of Brain Disorders, Collaborative Innovation Center for Brain Disorders, Capital Medical University, Beijing, China; ^5^Beijing Key Laboratory of Neuromodulation, Beijing, China

**Keywords:** magnetoencephalography, intracranial electroencephalogram, epilepsy, resting-state functional connectivity, surgical outcome

## Abstract

Epilepsy is caused by abnormal electrical discharges (clinically identified by electrophysiological recording) in a specific part of the brain [originating in only one part of the brain, namely, the epileptogenic zone (EZ)]. Epilepsy is now defined as an archetypical hyperexcited neural network disorder. It can be investigated through the network analysis of interictal discharges, ictal discharges, and resting-state functional connectivity. Currently, there is an increasing interest in embedding resting-state connectivity analysis into the preoperative evaluation of epilepsy. Among the various neuroimaging technologies employed to achieve brain functional networks, magnetoencephalography (MEG) with the excellent temporal resolution is an ideal tool for estimating the resting-state connectivity between brain regions, which can reveal network abnormalities in epilepsy. What value does MEG resting-state functional connectivity offer for epileptic presurgical evaluation? Regarding this topic, this paper introduced the origin of MEG and the workflow of constructing source–space functional connectivity based on MEG signals. Resting-state functional connectivity abnormalities correlate with epileptogenic networks, which are defined by the brain regions involved in the production and propagation of epileptic activities. This paper reviewed the evidence of altered epileptic connectivity based on low- or high-frequency oscillations (HFOs) and the evidence of the advantage of using simultaneous MEG and intracranial electroencephalography (iEEG) recordings. More importantly, this review highlighted that MEG-based resting-state functional connectivity has the potential to predict postsurgical outcomes. In conclusion, resting-state MEG functional connectivity has made a substantial progress toward serving as a candidate biomarker included in epileptic presurgical evaluations.

## Introduction

Epilepsy is a neurological disorder that is predominantly characterized by a tendency for the recurrent and unpredictable hypersynchronous neuronal activity that interrupts the normal brain function (Fisher et al., 2005). Antiepileptic drug administration is the first and basal treatment principle, but mediation does not work well for approximately one-third of epileptic patients who require epilepsy surgery evaluation that is aimed at localizing the epileptogenic zone (EZ) for individual surgical operations (Kwan et al., 2011). In current clinical practice, ictal activities and interictal epileptiform discharges (IEDs) are widely applied to localize the EZ (Nissen et al., 2016a; Cuello-Oderiz et al., 2018); however, some routine examinations are incapable of capturing ictal activities and occasionally even fail to acquire IEDs (Feyissa et al., 2017). Moreover, epileptic patients suffer from persistent seizures after the surgical removal of the EZ, potentially suggesting that the EZ in these patients is insufficiently identified and removed (Englot et al., 2015; Jobst and Cascino, 2015). Therefore, new approaches and biomarkers that can be integrated into clinical decision-making are urgent.

Epilepsy has been treated as an archetypical neural network disorder (Kramer and Cash, 2012), with specific disruptions in networks involving one or both hemispheres; consequently, the concept of epilepsy has changed from “foci” to “network” (van Diessen et al., 2013). Network analysis has been widely utilized to gain an insight into the dynamic complexity of refractory epilepsy, which provides a framework to describe seizure progress, including preseizure, seizure initiation, spread, and termination (Kramer et al., 2008; Schindler et al., 2008), and even interictal information (Chavez et al., 2010; Horstmann et al., 2010; Liao et al., 2010; van Dellen et al., 2012b). Recent studies have reported altered networks reflecting neuropathology in epilepsy patients with focal epilepsy (Coito et al., 2016a,b; van Diessen et al., 2014; Englot et al., 2016) and generalized epilepsy (Chavez et al., 2010; Zhang et al., 2011; Elshahabi et al., 2015; Niso et al., 2015). Resting-state networks are one of the common approaches in seizure surgical clinical evaluation to uncover the intrinsic interactions between brain areas that may affect epileptic networks, whose main advantage is that they can be estimated using interictal activity without the need to wait for a seizure to occur (Hsiao et al., 2015; Krishnan et al., 2015; Li Hegner et al., 2018; Leng et al., 2020).

**Figure 1:**
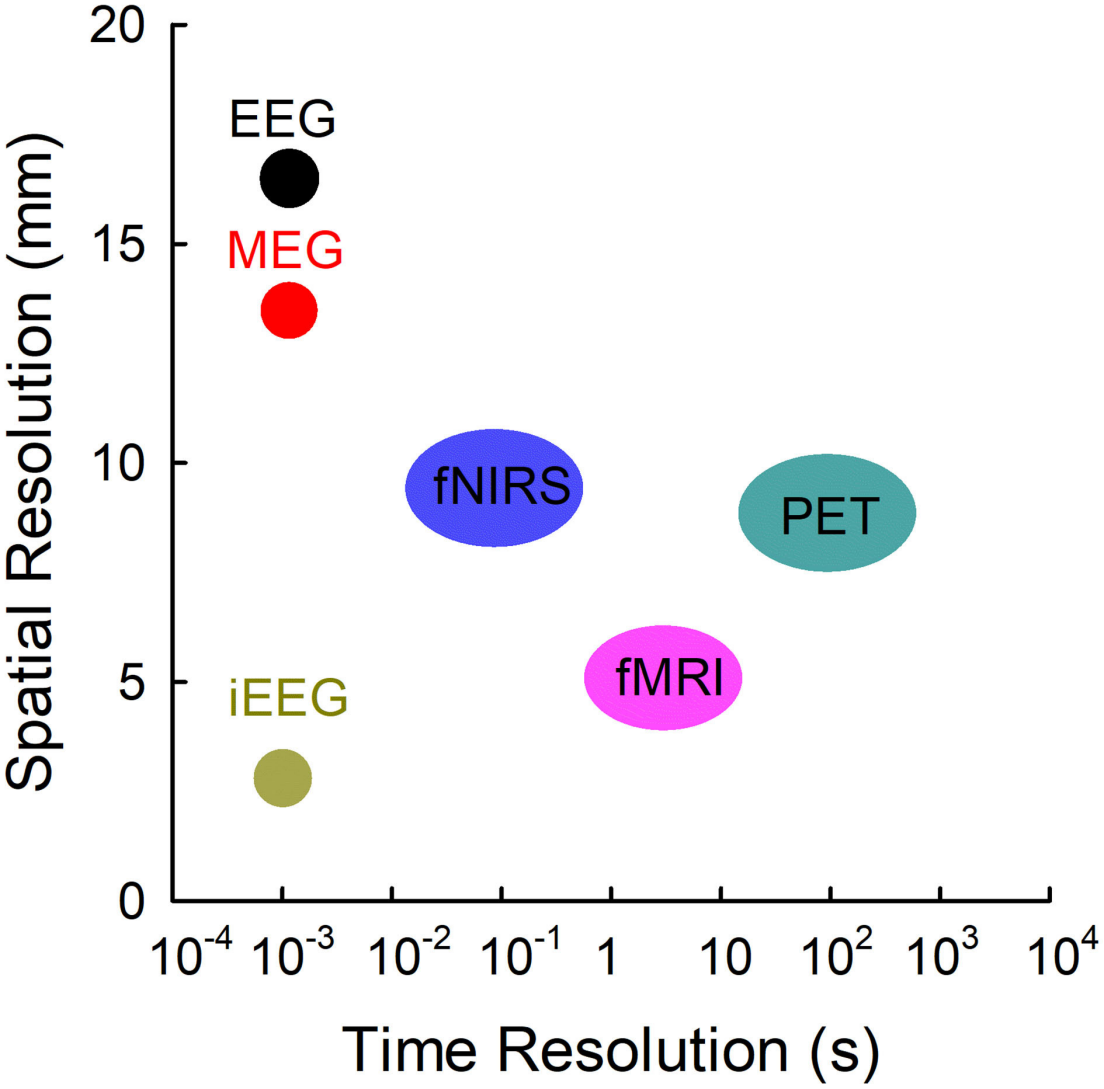
Temporal resolutions and spatial resolutions of different modalities commonly employed. EEG, electroencephalography; fMRI, functional MRI; fNIRS, functional near-infrared spectroscopy; iEEG, intracranial electroencephalography; MEG, magnetoencephalography; PET, positron emission tomography. Adapted from Olivi (2011).

Resting-state functional connectivity is attracting an increasing amount of attention as a tool for the surgical evaluation of epileptic patients. Advances in neuroimaging have generated multimodal technologies to analyze the resting-state functional networks (Figure 1). Of these imaging modalities, functional magnetic resonance imaging (fMRI) has been widely employed to investigate the functional connectivity during the resting state (Biswal et al., 1995; Palacios et al., 2013; Tracy and Doucet, 2015; Chakraborty et al., 2020; Courtiol et al., 2020). However, fMRI is an indirect measure of neural activity with a delay that detects the changes in blood oxygenation using blood-oxygen-level-dependent (BOLD) contrast (Scarapicchia et al., 2017). Similar to fMRI, positron emission tomography (PET) measures the blood flow by averaging the measurements with a low temporal resolution over a few minutes. Functional near-infrared spectroscopy (fNIRS) has limited spatial and temporal resolution. Electrophysiological neuroimaging modalities include scalp electroencephalography (EEG), intracranial electroencephalography (iEEG), and magnetoencephalography (MEG). Scalp EEG measures differences in voltage; its recordings depend heavily on the selection of the reference channel, and its activities are vulnerable to skull impedance. Unlike scalp EEG, MEG performs direct measures of brain activity at a specific point, and its measures are reference-free. In terms of localization accuracy, both MEG and scalp high-density EEG, through magnetic and electric source imaging, provide a good source localization of epileptogenic foci but with different sensitivities (Carrette and Stefan, 2019, Tamilia et al., 2019), and their combination showed complementarity due to the sensitivity of EEG and MEG for radial and tangential sources in the brain (Carrette and Stefan, 2019). Different from MEG and scalp EEG, long-term iEEG monitoring, mainly including electrocorticography (ECoG) and stereotactic electroencephalography (sEEG), is not only invasive and costly but also limited to a spatial sampling of only a portion of brain areas (Hader et al., 2013). MEG is a non-invasive technique that provides direct access to the entire brain activity with submillisecond temporal resolution and millimeter spatial resolution (Baillet, 2017), which makes it an ideal tool for investigating the resting-state functional connectivity in epilepsy.

## Basic Principles of MEG Measuring Brain Activities

**Figure 2:**
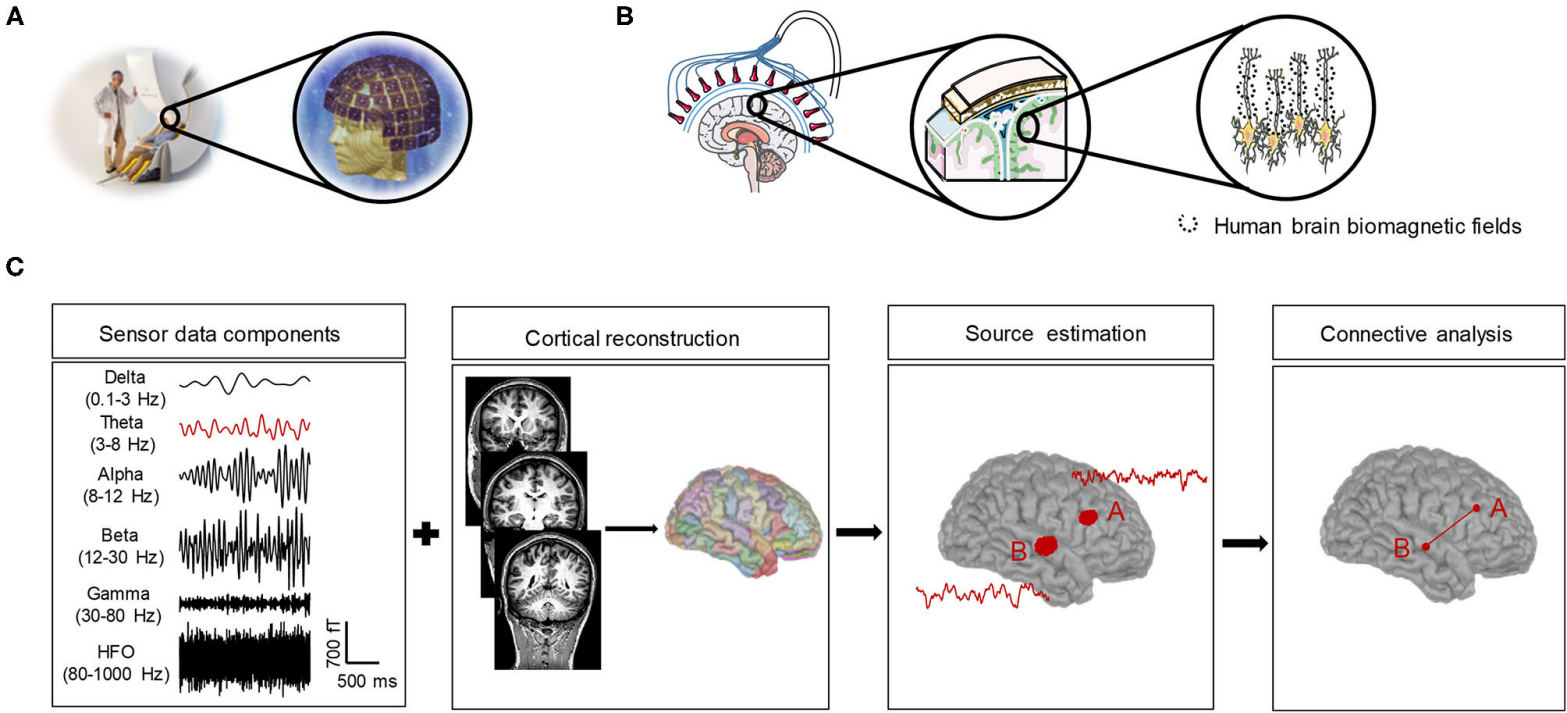
A simple overview of MEG and its measures and the pipeline to obtain the resting-state functional connectivity. **(A)** The subject sits on the MEG chair for the whole measurement process, whose head position corresponds to the sensors arranged in helmet-like arrays. **(B)** Some cortical pyramidal neurons are spatially aligned and perpendicular to the cortical surface. When these pyramidal neurons are excited, the apical dendritic membrane becomes transiently depolarized, which consequently triggers the generation of a current that flows from the apical dendrites to the soma within the intracellular space (primary current), and the magnetic field-generated intracellular currents are the sources of the MEG. **(C)** Pipeline to obtain the resting-state functional connectivity. Brain activities characterize several oscillatory bands (delta, theta, alpha, beta, gamma, and HFO), and each frequency-band signal (e.g., theta-band shown in red in the figure) of MEG sensor data is source-localized to parcellation atlases coregistered with anatomical MRI. Cortical regions with significant activity were considered ROIs **(A,B)**. The time-series activity of the ROI can be extracted using a beamformer and fed in the connectivity analysis to obtain the functional connectivity in the source space.

MEG measures the magnetic fields that are mainly generated by synchronous postsynaptic (intracellular) currents in the pyramidal neurons of the cerebral cortex (Hämäläinen et al., 1993). Some cortical pyramidal neurons are spatially aligned and perpendicular to the cortical surface, with the soma at the basal cortex and the apical dendrites at the surface of the cortex, which are the sources of magnetic fields that could be detected by MEG. When these pyramidal neurons are excited, the apical dendritic membrane becomes transiently depolarized, which consequently triggers the generation of a current that flows from the apical dendrites to the soma within the intracellular space (primary current), as well as the extracellular volume current that flows from the soma and basal dendrites to the apical dendrites. MEG signals are believed to arise from intracellular currents (Figures 2A,B).

Neuronal magnetic fields are considerably weaker by ~10–100 million times Earth's magnetic field (Hämäläinen et al., 1993), which highlights the need for MEG instrumentation with very sensitive sensors and the noise reduction method. In modern MEG systems, weaker fields are recorded using superconducting quantum interference devices (SQUIDs) (Kleiner et al., 2004), which are immersed in a liquid helium cooling unit set to ~-269°C that is highly sensitive to extremely subtle changes in the electromagnetic fields generated by neurons located a few centimeters from the sensors. In terms of noise reduction, in addition to the magnetically shielded room and differential (gradiometric) sensors that shield the MEG system from outside noise (Parkkonen, 2010), signal processing methods can effectively reduce noise. For example, signal space separation (SSS) decomposes multichannel MEG data based on the physical properties of magnetic fields to remove external disturbances and movement artifacts (Taulu et al., 2004). The signal space projection (SSP) uses the orthogonal projection method in the multidimensional signal space to remove the artifact associated with the spatial pattern of the magnetic field (Ramírez et al., 2011). Independent component analysis (ICA) is primarily employed to remove artifacts, such as blinking, eye muscle movement, facial muscle artifacts, and cardiac artifacts, but with poor resolution of highly correlated brain sources due to its fundamental statistical independence (Ikeda and Toyama, 2000; Iversen and Makeig, 2014).

## Resting-State Functional Connectivity Based on MEG Signals

Resting-state functional connectivity refers to the statistical associations or temporal correlations between two or more anatomically distinct brain regions without imposed stimuli. Although there is no established standard as to which method, modality, and analysis variant are optimal for MEG-derived, resting-state, functional connectivity, recent studies revealed methodological limitations by comparing the test–retest reliability (Colclough et al., 2016; Garcés et al., 2016), which provides a platform for inferring the connectivity of the resting state.

To calculate the resting-state functional connectivity, the activity of the cortical source responsible for MEG sensor signals should be estimated (Figure 2C). Each MEG sensor measures the signals generated by all brain sources that are active at a given time but with different weights due to the volume conduction effect (Winter et al., 2007). Given that the same connectivity information can be generated by different configurations of interacting sources, it is difficult to interpret the underlying functional connectivity based on MEG sensor signals. In addition, the head of the subject is not fixed relative to MEG sensor locations, which may cause the same MEG sensors from picking up signals from different regions of the brain for the same subject between two different runs of acquisition and different subjects with different head shapes, sizes, and positions under the helmet.

It is important to choose a suitable method for reconstructing the activity of brain regions from MEG sensor signals (Tadel et al., 2011; Xiang et al., 2014; Baillet, 2017; van Mierlo et al., 2019), which could be generally divided into two steps. In the first step, head modeling (or forward modeling) provides the mathematical relationship between the magnetic field of MEG sensors and the brain currents in a subject's head. The head model depends on the shape and conductivity of the head and can typically be constructed from structural MRI scans using methods ranging from a single sphere (Baillet et al., 2002) to overlapping spheres (Huang et al., 1999) and boundary or finite element methods (Darvas et al., 2004). Because the propagation of magnetic fields is not affected by electric conductance, the simple spherical head model often works quite well for the MEG forward model (de Munck et al., 2012; Hämäläinen et al., 2020).

In the second step, source modeling imaging depends on the head model to estimate neuronal activity from the MEG. Source model estimation of brain activity is typically obtained using two main approaches: (1) dipole modeling, where the position and amplitude of one to a few equivalent current dipoles (ECDs) are estimated over relatively short time windows (Leahy et al., 1998); and (2) distributed source modeling (DSM), which is aimed at estimating the activity in many sources in the brain by discretizing the brain (Baillet, 2017). In the ECD model, the sources of the MEG signals are assumed to consist of focal activations (Hämäläinen et al., 1993) and can be determined using non-linear optimization algorithms (Huang et al., 1998) or subspace scanning techniques (Mosher et al., 1992). ECDs are suitable and traditionally utilized in a clinic for epileptic focal localization (Bagic et al., 2011). Compared with ECDs, DSM may be more appropriate for source estimation when MEG signals are generated in a widespread manner (Hämäläinen and Ilmoniemi, 1994). The DSM approaches mainly include the weighted minimum norm estimate (MNE) (Hämäläinen and Ilmoniemi, 1994), standardized low-resolution electromagnetic tomography (sLORETA) (Pascual-Marqui, 2002), dynamic statistical parametric mapping (dSPM) (Dale and Sereno, 1993), maximum entropy on the mean (MEM) (Amblard et al., 2004), and beamforming and scanning methods (Liuzzi et al., 2017; Dimitriadis et al., 2018), each of which differs in their assumptions about cortical generators. The spatial fidelity of these approaches has been compared (Samuelsson et al., 2021). Although there is no optimal choice for the inverse solution, it is believed that the fidelity depends on the spatial and synchronization profiles of the interacting cortical sources (Hincapié et al., 2017).

Currently, although a large number of source–space connectivity estimation methods for MEG are available, there is no consensus on which functional connectivity index is most suitable for MEG resting-state studies. According to the logic of how to define the statistical dependence of the estimation source, connectivity methods can be divided into phase synchronization-based measures, coherence-based measures, generalized synchronization-based measures, and Granger causality-based measures (Niso et al., 2013). By comparing the consistency and reproducibility of some commonly employed network estimation metrics, Colclough et al. (2016) found that amplitude envelope correlation (AEC) and partial correlation are the most consistent methods, while the poorly consistent methods are phase-based or coherence-based metrics such as the phase lag index (PLI) or the imaginary part of coherency. Envelope correlation metrics for the resting-state MEG functional connectivity have also been recommended by Garcés et al. (2016) and have been widely applied in the connectivity network analysis (Brookes et al., 2011; Hipp et al., 2012; Dimitriadis et al., 2018; Aydin et al., 2020; Routley et al., 2020). The metrics, including coherence (Coh), imaginary coherence (imCoh), pairwise phase consistency (PPC), phase-locking value (PLV), PLI, weighted phase lag index (wPLI), and weighted phase lag index debiased (wPLI2), were compared in a recent study to test the reliability of resting-state MEG functional connectivity in schizophrenia (SZ). The article indicated that the reliability of these metrics varied greatly depending on the frequency band, network, and participant group examined (Candelaria-Cook and Stephen, 2020). Although there is no uniform standard for MEG resting-state functional connectivity, some identified factors should be considered: metrics (Colclough et al., 2016), frequency band (Meng and Xiang, 2016; Marquetand et al., 2019), and measurement duration (Marquetand et al., 2019).

In practice, to facilitate the source connectivity analysis of MEG signals, several open-source applications are available to the user (Table 1), for example, Brainstorm (Tadel et al., 2011; Niso et al., 2015, 2019), eConnectome (He et al., 2011), FieldTrip (Oostenveld et al., 2011), MNE (Gramfort et al., 2014), and SPM (Litvak et al., 2011), which provide a platform for the standardization of the most common analysis processes and reduce sharing efforts across MEG communities.

**Table 1:** Connectivity metrics utilized by open-source applications.

**Software**	**Connectivity metrics used**
Brainstorm	Amplitude envelope correlation Bivariate granger causality Correlation Coherence Phase locking value Phase transfer entropy
eConnectome	Adaptive directed transfer function Directed transfer function
FieldTrip	Coherence Directed transfer function Granger causality Imaginary part of coherency Partial directed coherence Phase locking value Phase slope index
MNE	Coherence Imaginary coherence Phase lag index Phase locking value Pairwise phase consistency Weighted phase lag index
SPM	Dynamic causal modeling

## Resting-State MEG Functional Connectivity Abnormalities Indicate Epileptogenic Networks

In recent years, several studies have shown that the network connectivity of the resting state in epilepsy has changed but with inconsistent results. These differences are probably attributed to the frequency band selected and the metric chosen to reconstruct the functional connectivity, as previously described, the different types of epilepsy investigated, and the different drug treatments and seizure rates in a clinic. Previous studies have reported that the resting-state functional connectivity is frequency-dependent when EEG and fMRI are applied (Samogin et al., 2020; Xia et al., 2020) and that resting-state functional connectivity derived from MEG exhibits significant changes with age from childhood to adolescence (Meng and Xiang, 2016). Moreover, the low- and high-frequency bands may represent different seizure networks (Tenney et al., 2014). Thus, we will focus on the evidence of functional connectivity in low- and high-frequency band abnormalities in epilepsy.

The activity of brain regions is characterized by several oscillatory bands with frequencies ranging from ~0.05 to 500 Hz (Buzsáki and Draguhn, 2004). As noted, MEG has shown advantages in connectivity metrics, which can exploit the rich frequency content of the MEG signal (Xiang et al., 2014; Baillet, 2017) by assessing the functional connectivity of oscillatory activities. The frequency-specific functional connectivity related to the different properties of the physical architecture of neuronal networks and to the speed of neuronal communication limited by axon conduction and synaptic delays should be considered. Based on this basic principle, the resting-state MEG functional connectivity derived from multifrequency oscillations is summarized in Table 2 and described in the next section.

**Table 2:** Resting-state MEG functional connectivity based on multifrequency signals recorded from epilepsy.

**References**	**Population**	**Frequency bands analyzed**	**Connectivity measure**	**Main findings**
Aydin et al. (2020)	Focal epilepsy	Theta (4–8 Hz), alpha (8–13 Hz), and beta (13–26 Hz) bands	Amplitude envelope correlation	Stronger functional connectivity in **alpha band** for non-seizure-free patients than seizure-free patients of post-surgical
Douw et al. (2010)	Gliomas with and without seizures	Delta (0.5–4 Hz), theta (4–8 Hz), lower alpha (8–10 Hz), upper alpha (10–13 Hz), beta (13–30 Hz), and gamma (30–45 Hz)	Phase lag index	Increased functional connectivity in the **theta** band related to higher seizure frequency in gliomas.
Englot et al. (2015)	Focal epilepsy Healthy controls	Delta (1–4 Hz), theta (4–8 Hz), alpha (8–12 Hz), beta (12–30 Hz), and gamma (30–55 Hz)	Imaginary part of coherency	Focal epilepsy vs. healthy controls: decreased connectivity in **delta, theta, alpha, and beta** bands
Hsiao et al. (2015)	Temporal lobe epilepsy (TLE) Healthy controls	Delta (1–4 Hz), theta (4–8 Hz), alpha (8–13 Hz), beta (13–25 Hz), and gamma (25–40 Hz)	Imaginary part of coherency	TLE vs. healthy controls: increased functional connectivity in **delta and theta** bands
Jeong et al. (2014)	Focal cortical dysplasia (FCD) Healthy controls	Theta (4–7 Hz), alpha (8–12 Hz), beta (13–30 Hz), and gamma (31–45 Hz) bands	Mutual information	FCD vs. healthy controls: increased connectivity in **beta and gamma** bands;
Leng et al. (2020)	Cingulate gyrus epilepsy Healthy controls	Alpha (8–13 Hz), beta (14–30 Hz), and gamma (31–80 Hz) bands	Correlation	Cingulate gyrus epilepsy vs. healthy controls: increased connectivity in **alpha, beta** bands, especially in **gamma** band
Li Hegner et al. (2018)	Focal and generalized epilepsy Healthy controls	Delta, theta, alpha, beta1, beta2, gamma	Imaginary part of coherency	Focal epilepsy vs healthy controls: increased connectivity in **theta, alpha** and **beta1** bands; Generalized epilepsy vs healthy controls: increased connectivity in **theta, alpha, beta1, and gamma** bands
Martire et al. (2020)	Temporal lobe epilepsy (TL) and temporal-plus(TL+) epilepsy	Theta, alpha, beta and low gamma	Phase lag index	TL vs. TL+: significant different connectivity of bitemporal and frontotemporal in the **theta, alpha, and beta bands**
Niso et al. (2015)	Focal and generalized epilepsy Healthy controls	Delta (0.1–4 Hz), theta (4–8 Hz) Hz, alpha (8–12 Hz) Hz, beta1 (12–20 Hz), beta2 (20–28 Hz), and low gamma (28–40 Hz)	Phase locking value	Focal epilepsy vs healthy controls: increased connectivity in **delta, theta, and beta1** bands; Generalized epilepsy vs healthy controls: increased connectivity in **delta, theta, alpha, beta1, beta2, and gamma** bands
Pourmotabbed et al. (2020)	Focal epilepsy with left- or right-hemisphere Healthy controls	Delta (0.5–3 Hz), theta (4–7 Hz) Hz, alpha (8–13 Hz) Hz, low beta (13–20 Hz), high beta (20–30 Hz), and low gamma (30–50 Hz)	Phase lag index	Focal epilepsy with right-hemisphere vs healthy controls: increased connectivity in **theta** band
Routley et al. (2020)	Juvenile myoclonic epilepsy (JME) Healthy controls	Delta (1–4 Hz), theta (4–8 Hz), alpha (8–13 Hz), beta (13–30 Hz), and gamma (40–60 Hz)	Correlation	JME vs. healthy controls: increased connectivity in the **theta** band and decreased connectivity in the **beta** band
van Dellen et al. (2012b)	Epilepsy with low-grade(LGG), high-grade glioma (HGG) and with non-glial lesions (NGL) Healthy controls	Delta (0.5–4 Hz), theta (4–8 Hz), lower alpha (8–10 Hz), upper alpha (10–13 Hz), beta (13–30 Hz), lower gamma (30–45 Hz) and higher gamma (55–80 Hz)	Phase lag index	LGG (NGL) vs. healthy controls: decreased connectivity in **theta** band
van Dellen et al. (2012a)	Lesional epilepsy	Delta (0.5–4 Hz), theta (4–8 Hz), lower alpha (8–10 Hz), upper alpha (10–13 Hz), beta (13–30 Hz), and lower gamma bands (30–48 Hz)	Phase lag index	Increased functional connectivity in the lower **alpha** band correlated with increased seizure frequency in regions where lesions were located.
Nissen et al. (2016b)	Focal epilepsy	Delta (0.5–4 Hz), theta (4–8 Hz), lower alpha (8–10 Hz), upper alpha (10–13 Hz), beta (13–30 Hz), gamma (30–48 Hz), broadband (0.5–48 Hz), and HFO (80–250 Hz) bands	Phase lag index	Concordance between **HFO** and spike sources
Meng (2019)	Epilepsy Healthy controls	Ripple (80–250 Hz), fast ripples (FRs, 250–500 Hz), and very high frequency oscillations (VHFO, 500–1,000 Hz)	Phase lag index	Epilepsy vs. healthy controls: higher mean functional connectivity in the **ripple and FRs** bands; Mean functional connectivity in the **ripple and VHFO** bands positively correlated with the duration of epilepsy
Yin et al. (2020)	Insular epilepsy Healthy controls	Ripples (80–250 Hz)	Correlation and Granger causality	Insular epilepsy vs. healthy controls: altered effective connectivity in interictal **HFO**

## High-Frequency Oscillations (HFOs)

Brain activities consisting of at least four clearly continuous oscillations with frequencies >80 Hz, compared with the background, are referred to as high-frequency oscillations (HFOs) (Jacobs et al., 2012; von Ellenrieder et al., 2016; Tamilia et al., 2017). The detection of HFOs is conventionally performed in an invasive intracranial EEG from patients with drug-resistant epilepsy (Akiyama et al., 2005; Jirsch et al., 2006; Urrestarazu et al., 2006; Fuertinger et al., 2016; von Ellenrieder et al., 2016; Qi et al., 2020; Ren et al., 2020; Zhao et al., 2020), whereas recent advances in MEG have shown promising results for non-invasive detection of HFOs in epilepsy patients (Miao et al., 2014; Nissen et al., 2016b; van Klink et al., 2016; von Ellenrieder et al., 2016; Meng, 2019; Yin et al., 2019). According to previous studies (Bragin et al., 1999; Usui et al., 2010, 2015; Brázdil et al., 2017), HFOs are further subclassified into three groups: ripples (80–250 Hz), fast ripples (250–500 Hz), and a very-high-frequency oscillation (VHFO) band (500–1,000 Hz) (Meng, 2019).

Proof of the validity of higher HFO rates within the seizure-onset zone (SOZ) has proposed HFOs as a promising biomarker of localization of the EZ (Thomschewski et al., 2019), which is attracting an increasing number of researchers to investigate HFOs in epilepsy with the goal of providing helpful information for a comprehensive preoperative evaluation. In general, HFOs are considered to be more focal and specific for identifying the SOZ than classical epileptic spikes (Jacobs et al., 2008, 2012; Andrade-Valença et al., 2012). Moreover, in regard to the identification of the SOZ, ripples have higher sensitivity but lower specificity than FR (Andrade-Valença et al., 2012). It seems that ripples and FR are generated by different pathophysiological mechanisms: Ripples are likely generated by synchronous firing coordinated by inhibitory currents (Schönberger et al., 2014), whereas FR may reflect the in-phase and out-of-phase firing of different pyramidal cell clusters (Demont-Guignard et al., 2012). Better results regarding the outcome prediction were reported for VHFO than for ripples and FR (Brázdil et al., 2017). However, there are still challenges regarding HFOs that need to be carefully addressed. One such issue is the detection of HFOs that are characterized by varied morphometry (Zijlmans et al., 2017), which should be addressed by exploiting an appropriate method to precisely detect HFOs (Ren et al., 2018; Zhao et al., 2020), considering the time-consuming and non-objective visual marking of HFOs. Another issue involves distinguishing pathologic HFOs from physiologic HFOs, representing a fundamental challenge for evaluating the effectiveness of HFOs as an epileptic biomarker (Thomschewski et al., 2019). Physiologic HFOs in the hippocampus and the occipital lobe of humans are a common phenomenon (Melani et al., 2013). Various analytical methods based on amplitude, frequency, and duration have been developed to separate HFOs (Cimbalnik et al., 2018; Thomschewski et al., 2019). Recently, Liu et al. (2018) identified a waveform morphological difference between pathologic HFOs and physiologic HFOs. Specifically, pathologic HFO waveforms are highly similar to those localized within the SOZs, whereas physiologic HFOs in random waveforms belong to the functional regions. An oddball cognitive task can be employed to further facilitate the discrimination of HFOs generated by epileptic and non-epileptic hippocampi (Pail et al., 2020).

**Figure 3:**
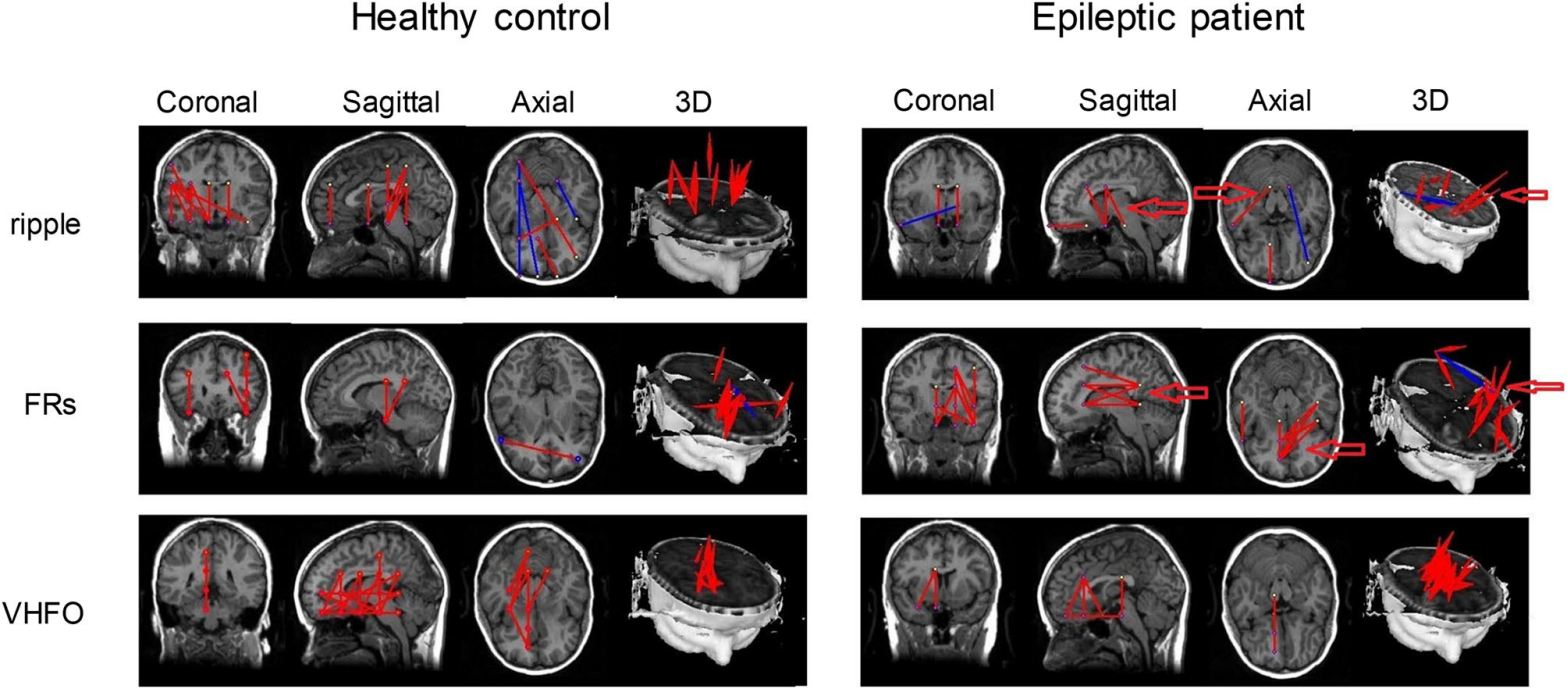
Functional connectivity networks based on HFOs from a healthy control and an epileptic patient. Altered connectivity patterns in the ripple and FR bands were found in epileptic patients. Arrows point to the significantly altered regions between epileptic patients and healthy controls. Inhibitory connections and excitatory connections are shown as blue lines and red lines, respectively. Adapted from Meng (2019).

A large number of studies have focused on the biomarker value of HFOs in the localization of the EZ (Zijlmans et al., 2012; Tamilia et al., 2017, 2020; Thomschewski et al., 2019; Velmurugan et al., 2019), whereas only a few studies have investigated the functional connectivity in HFOs using the resting-state MEG signals. Nissen et al. (2016b) recorded the resting-state MEG signals from 12 patients with refractory epilepsy and reported the enhanced functional connectivity in HFOs in the affected hemisphere compared to the non-affected hemisphere. However, Nissen et al. (2016b) analyzed HFOs in virtual MEG electrodes belonging to the sensor lever, which may include background noise. Using the accumulated source imaging method, Meng (2019) sourced HFO activities, reconstructed the brain network modulated by HFOs, and revealed that the brain networks of epileptic patients displayed altered patterns compared to those of healthy controls, especially within the ripple and FR bands (Figure 3). A recent study by Yin et al. (2020) indicated that patients with insular epilepsy showed an altered effective connectivity network in HFOs recorded from the resting-state MEG in contrast to healthy subjects.

## Low-Frequency Signals

Brain rhythms (<80 Hz) can be divided into five types: delta band (0.1–4 Hz), theta band (4–8 Hz), alpha band (8–12 Hz), beta band (12–30 Hz), and gamma band (30–80 Hz), which are referred to as “low-frequency signals” relative to HFOs (Xiang et al., 2014; Meng, 2019). Each type represents a different meaning in the neural system; for example, the delta band predominates during slow, deep, dreamless sleep, or pathological states during wakefulness. The theta and alpha bands are functions of arousal, attention, working memory, and long-range interactions (Palva and Palva, 2011). During the local stimulus or saliency process, focal network activity will involve more beta and gamma bands (von Stein and Sarnthein, 2000). Connectivity analysis based on MEG signals at different frequency bands could reveal different resting-state patterns (Brookes et al., 2011; Hillebrand et al., 2012), which has the potential to provide additional information for the preoperative evaluation of epilepsy and link specific patterns to specific forms of epilepsy. For example, Hillebrand et al. (2012) performed source-based analysis from MEG data for 13 healthy participants using a PLI estimator. They revealed for the first time the frequency-dependent functional connectivity within the resting-state networks across the cortex: Alpha-band connectivity and beta band connectivity were confined to posterior areas and sensorimotor areas, respectively, while gamma band connectivity was in a more dispersed pattern.

Altered functional connectivity derived from the resting-state MEG was observed in both focal epilepsy patients and generalized epilepsy patients. Some studies have reported that increased resting-state connectivity correlated with epilepsy. Resting-state functional connectivity based on beta and gamma frequency bands in focal cortical dysplasia (FCD) patients is stronger than that of healthy controls (Jeong et al., 2014). One study by Hsiao et al. (2015) indicated that the resting-state functional connectivity within the default mode network at the delta and theta bands was reinforced in temporal lobe epilepsy. Li Hegner et al. (2018) compared MEG-based resting-state functional connectivity among focal epilepsy, generalized epilepsy, and healthy controls. Compared with healthy subjects, they found that focal epilepsy showed an increased connectivity within the theta, alpha, and beta1 bands over bilateral temporal, parietal, insula, and frontal regions and that generalized epilepsy showed an increased connectivity in the theta, alpha, beta1, and gamma bands over widespread bilateral temporal, parietal, insula, and medio-frontal regions. Moreover, beta2- and gamma-band functional MEG connectivity in bilateral mesio-frontal and motor regions was increased for generalized epilepsy patients compared with focal epilepsy patients (Li Hegner et al., 2018). In addition, an increased functional connectivity in the alpha band correlated with an increased seizure frequency in regions where lesions existed. In a recent study by Aydin et al. (2020), the functional connectivity in the alpha band was stronger for non-seizure-free patients than for seizure-free patients after surgery.

However, some studies have reported the decreased resting-state connectivity in epilepsy patients. Using the metric of the imaginary part of coherency to calculate the functional connectivity, Englot et al. (2015) found that focal epilepsy showed a decreased connectivity in delta, theta, alpha, and beta bands compared to the healthy controls. The increased alpha-band functional connectivity in the MEG resting-state network for low-grade glioma patients after resective surgery was suggested to be related to improved cognitive performance (van Dellen et al., 2012a). A recent study by Leng et al. (2020) investigated the functional connectivity of the default mode network in cingulate gyrus epilepsy using the resting-state MEG signals, analyzed multifrequency signals, and revealed that cingulate gyrus epilepsy has enhanced functional connectivity in alpha, beta, and gamma bands between the angular gyrus and the posterior cingulate cortex (PCC) of the left hemisphere compared to healthy controls. Moreover, the dominant functional connectivity in the gamma band over that in the alpha and beta bands indicates the significant contribution of the gamma band to the default mode network in cingulate gyrus epilepsy. Since the PCC is considered to be the only node in the default mode network that directly interacts with almost all other nodes (Fransson and Marrelec, 2008), the prominent functional connectivity in the gamma band between the PCC and left angular gyrus may be a potential biomarker for cingulate gyrus epilepsy.

**Figure 4:**
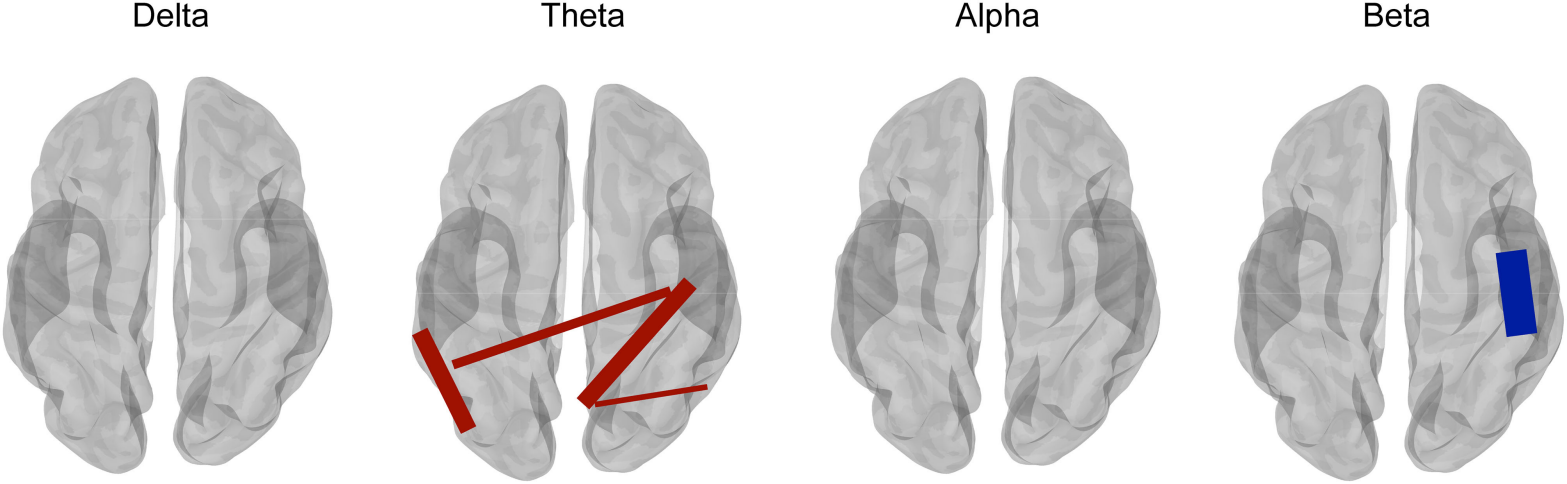
Comparison between juvenile myoclonic epilepsy (JME) patients and healthy controls for multifrequency bands with corrected *t*-tests. Compared with healthy controls, JME showed an increased connectivity in the theta band (4–8 Hz) (shown in red lines) and a decreased connectivity in the beta band (13–30 Hz) (shown in blue lines). However, connectivity in the frequency bands of delta (1–4 Hz) and alpha (8–13 Hz) did not show a significant difference with corrected *t*-test between JME and controls. Adapted from Routley et al. (2020).

In addition, complex patterns of increased and decreased connectivity were also reported in epilepsy. Juvenile myoclonic epilepsy (JME) is one of the most common epilepsy syndromes and is considered a brain network disorder with predominantly frontal (Chowdhury et al., 2014) but also parieto-occipital and subcortical involvement (Gotman et al., 2005). Routley et al. (2020) investigated the differences in the resting-state MEG functional connectivity using the AEC metric between patients with JME and healthy controls and found an increased connectivity in posterior theta and alpha bands and a decreased beta-band connectivity in sensorimotor brain regions in JME patients (Figure 4). The alterations in connectivity were similar to previous reports from EEG recordings in JME patients with an increased alpha-band connectivity and a decreased beta-band connectivity (Clemens et al., 2011). Theta and alpha bands have the function of attention and working memory, while patients with JME show cognitive dysfunction in attention and working memory (Pascalicchio et al., 2007). In addition, beta-band oscillation is considered to be involved in sensorimotor regulation (Engel and Fries, 2010). Routley et al. (2020) further suggested that the altered resting-state MEG connectivity may be the resting neurophysiological hallmark of JME.

In summary, the different changes reported here may be attributed to clinical differences, types of epilepsy, and methodology. Although the aim of linking specific-frequency patterns to specific types of epilepsy based on the MEG resting-state functional connectivity is a major challenge, it represents a promising method in the future and provides additional information. The metric and procedure used to calculate the functional connectivity need a unified standard, and more added information should be obtained by combining multimodal approaches.

## Multimodal Integration of MEG and iEEG

As previously described, the resting-state functional connectivity in the multifrequency band can be investigated by multimodal neuroimaging approaches. Integration of different technologies may provide complementary information to more precisely characterize epileptogenic networks. As a “gold standard,” iEEG, including ECoG and sEEG, provides the opportunity to directly detect activities of the deep regions of the brain but is limited to only a portion of brain areas. Accumulating studies have demonstrated that MEG provides a broad whole-head view of brain activities that further guide implantation sites for intracranial recordings (Knowlton et al., 2006; Grova et al., 2016). Therefore, the integration of MEG and iEEG approaches with the advantage of obtaining local and global activities of the brain should be considered to study the resting-state functional connectivity that supports clinical decision-making.

The correlation between MEG and iEEG performed on separate recordings in presurgical evaluation has been reported in previous studies (Oishi et al., 2002, 2006; Knowlton et al., 2009; Bouet et al., 2012; Almubarak et al., 2014; Grova et al., 2016; Murakami et al., 2016), and concordant evaluation based on MEG and iEEG is associated with more favorable surgical outcomes than inconsistent evaluation. A comparison of networks based on oscillatory activities and spikes in MEG and iEEG was made, indicating the presence of overlapping but different networks for oscillations and spikes, which provide complementary information for presurgical assessment (Jmail et al., 2016).

In addition to considering the correlation between MEG and iEEG data, another crucial method is to verify the technical and clinical aspects applied to simultaneous MEG and iEEG recordings (Santiuste et al., 2008; Dubarry et al., 2014; Gavaret et al., 2016; Badier et al., 2017). Moreover, simultaneous MEG and iEEG recordings increase the sensitivity for localizing the epileptogenic region (Kakisaka et al., 2012; Vivekananda et al., 2020). This approach offers unique opportunities for cognitive neurophysiology research. For instance, Crespo-García et al. (2016) demonstrated a negative correlation between slow-theta activities in the hippocampus and spatial memory accuracy using simultaneous MEG and iEEG methods. Therefore, it is expected that the resting-state connectivity analysis will benefit from multimodal simultaneous recordings of MEG and iEEG signals at multiple spatial scales in the future.

## MEG Resting-State Connectivity: Potential Value as a Predictor of Surgical Outcome

Seizure-free status is the evaluation criterion for the quality of life of patients with epilepsy. Although advancements in localizing the SOZ assist in clinical decision-making, approximately one-third of patients continue to experience postoperative seizures (Malmgren and Edelvik, 2017). Although the challenge is mainly attributed to heterogeneous patients and the complexity of brain network interactions, a correlation between the surgical outcome and the resting-state functional connectivity has been reported, as suggested mostly using fMRI (Tracy and Doucet, 2015; Englot et al., 2016; He et al., 2017; Morgan et al., 2019; Pressl et al., 2019). As a neuroimaging technology with the advantage of high spatial and temporal resolution, will MEG resting-state connectivity be a candidate predictor of surgical outcome in epilepsy?

The MEG resting-state connectivity reconstructed from the low-frequency oscillations has been assumed to be associated with surgical outcome. Using the metric of the imaginary part of the coherence on the resting-state MEG signals, Englot et al. (2015) demonstrated that patients with increased connectivity in the alpha band within the resected region were more likely to have favorable outcomes. However, inconsistent results have reported a connectivity in the alpha band between the brain regions where IEDs are generated and that the remainder of the cortex was weaker for seizure-free patients but stronger for non-seizure-free patients compared with the connectivity in the alpha band between the corresponding contralateral homologous region and the remainder of the cortex (Aydin et al., 2020). One possible reason for this finding involves the different metrics selected, i.e., imaginary part of the coherence and AECs, to calculate the resting-state functional connectivity. Several MEG studies have investigated the resting-state connectivity based on HFO signals (Nissen et al., 2016b; Meng, 2019; Yin et al., 2020) without investigating the correlation between HFO connectivity and postoperative outcomes. However, an increased directed connectivity in the ripple band was found in the resected area of patients with good postoperative outcomes using ECoG (Zweiphenning et al., 2019).

MEG network hubs, namely, regions with high network connectivity, are associated with the surgical outcome. Nissen et al. (2017) investigated whether MEG network hubs overlapped more with the resection area in seizure-free patients and found that hubs were localized within the area later resected in nine of 14 seizure-free patients and in none of eight patients who were not seizure-free. They subsequently investigated whether this overlap is distinct between seizure-free patients and non-seizure-free patients after surgery but found no significant difference in the overlap between the two surgery outcomes (Nissen et al., 2018). Moreover, a recent study investigated MEG network hubs and their removal in a cohort of 31 patients with refractory focal epilepsy and reported that seizure-free patients had more network hubs surgically removed than non-seizure-free patients (Ramaraju et al., 2020), which suggests that removing MEG network hubs may be a method for improving surgical outcomes. As a result, MEG resting-state connectivity should be considered a potential predictor of surgical outcome, which would be added to the presurgical evaluation of epilepsy.

## MEG Resting-State Connectivity in Eyes-Closed and Eyes-Open States

Considering that the subjects requested to keep their eyes closed (EC) or eyes open (EO) during the resting-state MEG scan, it is important to know whether the choice of keeping EC or EO affects the resting-state functional connectivity. Previous studies have demonstrated significant differences in the resting-state connectivity patterns between EC conditions and EO conditions using fMRI (Yan et al., 2009; van Dijk et al., 2010; Liu et al., 2013; Patriat et al., 2013; Wei et al., 2018; Agcaoglu et al., 2019, 2020; Weng et al., 2020). However, few studies have investigated the potential difference in the resting-state functional connectivity in the EC and EO states using MEG in the same manner as fMRI. Horstmann et al. (2010) analyzed the resting-state functional brain networks of epileptic patients and healthy controls from EEG and MEG data recorded in EC and EO conditions and indicated that functional networks in both groups are more regular during the EC state compared with the EO state, which is derived from the EEG recordings but hardly derived from the MEG recordings. Liu et al. (2010) reported the distinct alpha-band activities of MEG signals under EC and EO conditions. Jin et al. (2014) further investigated the brain functional network in the EC and EO conditions of the MEG resting state from 39 healthy subjects and found an enhanced functional connectivity in the theta and alpha bands during the EO state relative to the EC state. In a recent study, functional connectivity in the theta band was significantly different between healthy subjects in the EO state and focal epileptic patients in the EC state (Pourmotabbed et al., 2020); however, the role of eye behavior states was uncertain.

These significant differences in the resting-state functional connectivity between the EO state and the EC state may support the hypothesis of Marx et al. (2003) on mental states, including the “interoceptive” network, which was characterized by imagination and multisensory activity during EC, and the “exteroceptive” network, which can be characterized by the attention and ocular motor activity during the EO state. Both of these states constitute intrinsic brain activity. The alpha activity was assumed to be associated with eye states, and EEG and MEG were simultaneously employed to analyze the “alpha” functional network of the specific regions of the brain insusceptible to the different eye conditions. The functional connectivity of the distinct brain regions decreased over time (Anwar et al., 2014). Considering that the resting state with EO or EC might influence functional connectivity, performing an analysis using data from both states would add further information to this area of uncertainty.

## Conclusion

Epilepsy has been widely considered an archetypical brain network disorder that is commonly investigated using the resting-state functional connectivity. With its advantages of high temporal resolution and spatial resolution, MEG has been increasingly used to investigate the resting-state functional connectivity in epileptic presurgical evaluation. In this paper, the different metrics of abnormal functional connectivity are reviewed based on low-frequency signals and high-frequency oscillations. The application of MEG combined with iEEG approaches enhances the characterization of functional connectivity in the resting-state network, which provides complementary information for improving epilepsy surgery. More importantly, the resting-state functional connectivity based on MEG signals represents a potential predictor of postsurgical seizure outcome. Considering the resting-state connectivity differences between eyes-open conditions and eyes-closed conditions, an approach performing analysis using data from both states would be added to obtain more information about this area of uncertainty. The findings reviewed here provide grounds that the resting-state functional connectivity derived from MEG signals provides a strong contribution to a presurgical evaluation in epilepsy.

## Author Contributions

NX, WS, JQ, JW, and QW all contributed to the writing and revision of this manuscript. All authors contributed to the article and approved the submitted version.

## Conflict of Interest

The authors declare that the research was conducted in the absence of any commercial or financial relationships that could be construed as a potential conflict of interest.
